# The impact of leadership style, reward system, environmental strategy and environmental management accounting on environmental performance of Vietnamese manufacturers

**DOI:** 10.1371/journal.pone.0323662

**Published:** 2025-05-21

**Authors:** Vo Tan Liem

**Affiliations:** Faculty of Economics and Management, Van Hien University, Ho Chi Minh City, Vietnam; IUB: The Islamia University of Bahawalpur Pakistan, PAKISTAN

## Abstract

The Upper Echelons Theory (UET) was employed in this study to examine the effects of Green Transformational Leadership (GTL) on the Green Reward System (GRS), Proactive Environmental Strategy (PES), Environmental Management Accounting (EMA), and Environmental Performance (EP). The data was collected from 198 CEOs of Vietnamese manufacturing enterprises by using a survey technique. The hypotheses were tested by using SmartPLS3, and the results indicate that all hypotheses are statistically significant. This research made a theoretical contribution to UET by investigating the key roles of GTL, GRS, PES, and EMA in the attainment of environmental performance. The study’s results have suggested that top managers and organizational policymakers should implement the practices of the CEO’s GTL, as well as GRS, PES, and EMA, to address stakeholders’ environmental sustainability concerns and preserve their market position.

## 1 Introduction

In recent decades, the environment has been significantly impacted by excessive resource use and intensified industrial activities, which contribute to cumulative environmental pollution [1,2]. Organizations face considerable pressure from stakeholders to reduce hazardous waste and participate in environmental safety programs [3]. Manufacturing enterprises are increasingly pressured to prioritize environmental concerns [4,5]. Consequently, enterprises are experiencing significant pressure from many stakeholders to mitigate environmental impacts through their production and management practices [6]. Additionally, organizations are apprehensive about the financial repercussions, as adhering to their traditional procedures will result in the loss of investors and consumers [3]. In order to address this issue, they also require a substantial investment; however, the majority of organizations fail financially due to a lack of information and poor organization [7,8]. Numerous studies have been conducted to address these grievous issues, and the most recent research indicates that there is a greater emphasis on the utilization of sustainable resources and organizational sustainability [9,10]. Manufacturing firms are actively engaging in environmental sustainability initiatives and are increasingly involved in green human resource management, as well as environmental strategy and control systems, to foster a sustainable environment [11–13].

The quest for sustainable development is challenging because of considerable opposition to innovation [14]. The conservation of the green environment can be attained by cultivating a corporate culture that encourages innovation and endorses environmentally sustainable business practices [15]. Top leaders and followers must be willing to have their behavior evaluated against generally accepted societal values [16]. Fry [17,18] asserts that spiritual leadership is essential for an organization’s success in fostering and sustaining a learning culture, in conjunction with motivational, charismatic, or transformational leadership. According to Aslam, Yusof [19], transformational spiritual leaders maintain their vision in the face of errors, fear, confusion, or discontent; subsequently, they (leaders/followers) “transform” their ideas into the minds of others to support one another. Transformational leadership is a leadership style that provides vision and mission for the organization, as well as inspires pride, respect and trust from employees. Managers with a transformational leadership style are highly capable of communicating, directing employees to effort, and expressing important organizational goals in the simplest way; at the same time, they are able to promote employee creativity and problem-solving with care as well as give employees individual attention and advice [20]. Recently, multiple studies indicate that green transformational leadership (GTL) is crucial for fostering green innovation and enhancing the environmental performance of firms [3,5,16]. Top leaders with GTL style – through choices about vision, purpose, mission, strategy, and their implementation – are responsible for creating vision and value congruence across all organizational levels as well as developing effective relationships between the organization and environmental stakeholders [16]. Employees demonstrating eco-friendly behavior are more likely to engage in environmental sustainability through the adoption of innovative practices; thus, leadership should focus on fostering such behaviors among employees to successfully attain organizational environmental objectives [14]. GTL enhances the green innovation process and fosters environmental sustainability by offering essential inspiration and incentive to employees to meet designated organizational and environmental objectives [21]. Organizations utilize GTL to modify employee behavior in order to reduce industrial pollution, hence ensuring environmental sustainability and enhancing corporate environmental performance [22,23].

It is essential for today’s businesses to establish an organizational culture that prioritizes environmentally responsible behavior among employees, as green human resource management methods are essential for influencing employee behavior in contemporary organizations [14,24]. Researchers indicate that employees significantly impact the environmental performance of firms [25,26], but enhancing environmental performance necessitates a more prominent involvement from top management [1]. Furthermore, top management shapes the creation of an administrative framework applicable across the organization [27], while a green reward system (GRS) plays a crucial role in motivating employees and recognizing their substantial contributions to environmental management [28]. The aim of implementing reward criteria is to enhance, sustain, and incentivize employee performance while emphasizing the significance of environmental protection [29,30].

A leadership style also has a substantial impact on the decision-making process for selecting and implementing long-term environmental strategies for the organization as a whole [27]. Proactive environmental strategies (PES) are rarely applied in developing economies’ industries, yet they have the ability to significantly impact competitive advantage and sustainable development in industrial sectors [31,32]. Environmental management accounting (EMA) is an extremely effective control approach for improving the environmental performance of manufacturing enterprises [3]. EMA also helps firms overcome the limitations of traditional management accounting techniques [2,33]. EMA allows firms to establish environmentally friendly production techniques and procedures while reducing harmful waste [34].

To reduce the negative environmental effect, organizations employ green practices, formulate, and implement their work strategies to reduce waste, foster a green or healthy environment, conserve energy, and contribute to environmental sustainability [35]. According to Dai, Cantor [36], a firm can boost its EP when it coordinates its strategic environmental emphasis with its green supply chain integration mechanism. Liu and Zhang [37] examined the impact of EMA and green human resource management on green organizational behavior. However, these studies have not paid attention to management control systems to promote green organizational behavior; in addition, green organizational behavior needs to be measured specifically and clearly. Recently, Hanif, Ahmed [3] examined the impact of GTL style and EMA adoption on organizational environmental performance, but this research didn’t consider the potential impact of GRS and the role of PES on environmental performance. That is, they did not explore the impact of GTL style on the role of GRS and PES. This has overlooked the recognition in previous literature of the influence of leadership style on the choice of administrative systems in organizations as well as the role of top managers in making decisions on the choice of long-term environmental strategies that organizations pursue [27]. While the administrative systems and strategies implemented by organizations play a pivotal role in the success of improving the organization’s EP, this has been demonstrated in many previous empirical studies [2,3]. In addition, the needs and behaviors of top managers as well as the implementation of environmental strategies, implementation of control systems are different in each country due to differences in economy, culture and history... [38]. This study aims to address the gap in the current literature and extends the study of Hanif, Ahmed [3] by examining the impact of top managers’ GTL on GRS, PES, and EMA choices, as well as the impact of these factors on EP. In addition, this study will provide a better understanding of the benefits of using the EMA information in Vietnamese manufacturers. Furthermore, understanding the impact of related factors on top managers’ implementation of the EMA in Vietnamese manufacturers may help top managers to use this information more effectively in making decisions, which may in turn improve their EP. The research study aims to address the research questions:

RQ1. What are the effects of GTL on GRS, PES, EMA, and EP?
RQ2. What are the effects of PES on GRS, EMA, and EP?
RQ3. What are the effects of GRS on EP and EMA?
RQ4. What is the impact of EMA on EP?

This study identified substantial deficiencies in the current literature. This research model is primarily constructed by using UET, an overlooked framework in EMA. Previous research on EMA and employee community participation is usually based on the Natural Resource-Based View (NRBV) or stakeholder theory [3,39]. Therefore, this study contributes to the burgeoning body of research utilizing UET in the domains of EMA and GRS. EMA is seen as a sophisticated administrative framework within organizations [2]. This study further substantiates that EMA information is a crucial resource for Vietnamese manufacturing organizations in enhancing environmental performance. Furthermore, the study’s findings enhance the UET [27] by demonstrating the significance of PES and GRS in maintaining the firm’s environmental performance. The study’s conclusions indicate that GTL should be regarded as a strategic resource for manufacturing enterprises. Secondly, despite GTL’s significant influence on GRS, PES, EMA, and EP, less study has been conducted to examine the effect of these enablers in enhancing EP within the production sector [2,3]. Therefore, based on UET, this study establishes a connection among top managers’ GTL style, PES, control systems (GRS and EMA), and organizational environmental performance [27]. Moreover, this research has examined within a single study the impact of GTL on PES and the two complexities of administrative system implementation: green reward system and environmental management accounting, alongside reliance on environmental performance [14,40,41]. Third, the current literature indicates that green innovation is a broadly recognized phenomenon that assists firms in alleviating the negative impacts on the environment through sustainable practices [8,42]. Certain academics propose that companies should leverage intangible resources more effectively to confront environmental sustainability challenges and satisfy stakeholder concerns [1,26]. Appropriate focus is lacking on the application of a combination of GRS and EMA as effective support mechanisms in the execution of proactive environmental strategies and the enhancement of environmental performance [27]. Nevertheless, research on the relationships among these elements has not attracted much attention.

This study also indicates several management implications. Many organizations in developing countries are not implementing EMA to improve their environmental impact, and they remain unable to actively contribute to environmental sustainability but adhering to traditional accounting practices, which is why they struggle to address negative environmental consequences. This study assists top managers in manufacturing firms in recognizing the significance of GRS, PES, and the beneficial function of EMA information, so encouraging them to formulate and choose environmental systems and strategies inside their organizations. Secondly, in the execution of green innovation within manufacturing firms, top managers, particularly CEOs, serve as the most critical and essential aspect for the success of this initiative. This study enables top executives to alter their leadership approach regarding environmental considerations and to reassess the significance of GRS, PES, and EMA in facilitating green innovation processes within their organizations. Assist designers of GRS and EMA systems in securing the support from top managers.

## 2 Literature review and hypothesis development

### 2.1 Upper echelon theory

**Fig 1:**
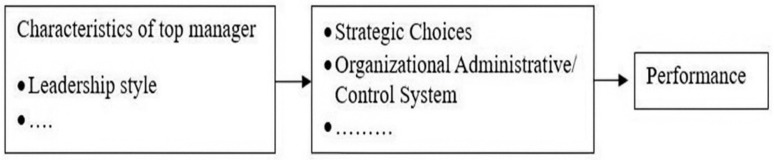
Conceptual model of upper echelons theory.

In the 1980s, following an examination of the focus of prominent global business publications on CEOs’ socio-demographic characteristics, Hambrick and Mason introduced a conceptual framework asserting that “organizations become reflections of their top managers” (1984, p. 193). Due to its historically changing and cross-fertilizing foundation, UET has been associated with several research topics both within and beyond the management domain over time [43]. This study illustrates that leadership style and other internal dispositions significantly affect critical decisions and results of organizations (Fig 1).

This study was motivated to address concerns raised by Hambrick and Mason [27], Hiebl [44] who posited that leadership style is an important but often overlooked factor when selecting management control systems to enable top management to communicate, empower, and implement their vision. Previous literature has found that the GTL style can develop and maintain a control and reward system that is oriented towards innovation and creativity related to improving environmental performance [3,8]. Therefore, studying the role of GTL style as an antecedent in the design and implementation of environmental control systems such as GRS and EMA, and GTL can provide important insights into the motivation for an organization’s choice of environmental strategies.

UET is considered an important foundational theory regarding the behavior of top managers [45]. UET has indicated that the choice of a strategy for the organization to pursue and implement is influenced by the leadership style of top managers [27]. Currently, GTL is a leadership style that is attracting the attention of many scholars [3,12]. Additionally, UET, after its development process, has focused on the leadership style of top managers influencing the implementation and use of complex administrative systems within organizations [2]. Hambrick and Mason [27] argue that control systems in general, and EMA and GRS in particular, are considered complex administrative systems, components in the organization’s output influenced by the leadership style of top managers. EMA and GRS are considered outputs or aspects of the organizational structure [2]. Therefore, EMA and GRS are considered a perfect and complex formal planning and measurement system within the structure related to environmental issues [2,14]. Hambrick and Mason [27] acknowledged that organizational control systems are established and built by top managers, and these control systems cannot be imposed on them. Therefore, based on UET, this study has sufficient grounds to examine the impact of the top manager’s GTL on the choice of PES strategy, the implementation of GRS, and EMA.

UET acknowledges that the leadership style of top managers affects the organization’s performance through the choices they make. Additionally, UET also confirms that an organization’s strategy (such as PES) or a complex administrative system (such as EMA and GRS) impacts the organization’s performance. Many empirical studies based on UET have also demonstrated that an organization’s EP is influenced by the characteristics of top managers, either directly or indirectly through the chosen environmental strategy or complex administrative systems [2,3]. Therefore, based on UET, this study has sufficient grounds to examine the impact of GTL, PES, GRS and EMA on EP.

**Fig 2:**
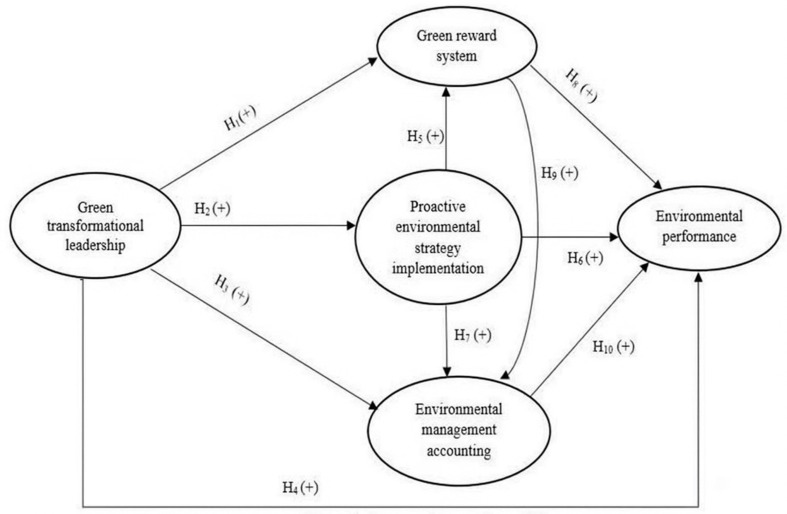
Proposed research model.

To test the above relationships, this study developed a model (Figure 2) that highlights the significant effect of GTL on EP, improving EP through GTL, GRS, and using EMA information by top managers. The proposed study would be helpful for top managers in dealing with environmental problems and developing control systems and strategies to reduce hostile impacts on the environment.

### 2.2 Proactive environmental strategy

The environmental strategies of organizations are categorized as proactive, reactive, accommodating, and defensive strategies [46]. Do and Nguyen [47] and Banerjee, Lyer [48] assert that, despite many classifications of environmental strategies ranging from reactive to proactive stages, emphasis should be placed on the commitment to environmental issues. Nonetheless, the reactionary strategies are generally mandatory, and the organization must comply with them. The research on environmental management indicates that proactive environmental strategy is a significant subject garnering growing attention [12,49]. An effective environmental strategy encompasses a collection of objectives, visions, plans, and processes designed to avert adverse environmental effects and exceed basic compliance with environmental rules [50]. An organization with a proactive environmental strategy can effectively utilize its tangible and intangible resources, resulting in diminished environmental impact, enhanced performance, and augmented competitive advantage [49]. Proactive environmental strategy may be categorized into two components: (1) strategy content and (2) strategy design and implementation [51]. Strategy design and implementation are critically important in environmental management, as a well-crafted plan and effective methods for achieving objectives enhance environmental performance [13].

### 2.3 Green transformational leadership

Vietnamese traditional core values have coexisted with cultural values from China, France and America, after a long history of being influenced by these countries and these mixed values influence the leadership style of Vietnamese top managers [52]. Transformational leadership is a leadership style defined by leaders that identify the necessity for change and adeptly motivate teammates to collaboratively strive towards a common objective [53]. Avolio, Bass [54] assert that team members are motivated and subordinates become more aware through the emphasis on elevated principles such as equity, justice, and liberty. GTL entails leaders inspiring and motivating their teams to attain environmental goals and surpass expected standards of environmental performance [55]. This leadership style promotes the prioritization of organizational objectives over individual aspirations, offers direction and support in many circumstances, and cultivates enthusiasm among employees to generate innovative concepts for the organization [8].

The effects of leadership style on managers’ use of the accounting information also depend on the context [38]. A top manager’s transformational leadership style plays an important role in creating organizational systems and motivating subordinates [56]. Top leaders play a significant role in innovate the company, particularly top leaders with GTL [57]. However, particular leadership styles have different effects on managers’ use of the EMA information in making decisions to improve environmental performance [2]. In addition, GTL is tasked with guiding and motivating its personnel to participate in environmentally sustainable innovation [58]. When top managers motivate and endorse novel concepts, present constructive challenges, and articulate an inventive vision, employees are more inclined to exhibit creativity and innovation [59]. Therefore, Vietnamese manufacturers need to understand the way GTL style may encourage top managers’ use of the EMA information, inspiring and motivating their employees through GRS to make decisions in order to achieve high environmental performance.

### 2.4 Green reward system

GRS refers to the alignment of the system with the environmentally sustainable policies and practices employed by the organization. It should be structured to implement sustainable efforts in the workplace and lifestyle, and to diminish carbon footprints [60]. Individuals ought to be incentivized with rewards for their commitment to comprehending and fostering an eco-friendly culture [61]. Numerous reward systems exist that organizations may employ to facilitate the acquisition of green skills. GRS also fosters the cultivation of employees’ environmentally conscious behavior [62]. Consequently, it is evident that several organizations have implemented reward structures to cultivate suitable employee attitudes awards are largely classified into monetary awards (e.g., bonuses, cash, rates) and non-monetary benefits, including vacations, leave, and presents for employees, as noted by Renwick, Redman [63].

In the Vietnamese centrally planned economy, the reward system was not directly related to organizational efficiency and individual efforts [64]. Appropriate rewards may have a positive effect on employees’ efforts [52]. In the same line, the GRS encompasses all economic and non-economic incentives offered to employees to attract, motivate, and involve them in attaining green corporate objectives [65,66]. Therefore, Vietnamese manufacturers need to understand a GRS are suitable for motivating and supporting top managers to make decisions in order to achieve high environmental performance.

### 2.5 Environmental management accounting

EMA is defined as the process of describing organizational procedures, comprehending environmental data, and translating occurrences to create common knowledge and conceptual patterns among top managers [67]. CEOs can use the EMA system to lessen the environmental effects of their business and enhance decision-making when faced with demands from outside sources [68]. CEOs may discover that EMA information can aid them in the identification, collection, utilization, and assessment of a diverse array of accounting information sources, thereby enabling them to make informed and advantageous decisions regarding their organization’s environmental management [69]. EMA combines economic accounting data with environmental data to demonstrate how the two are related and influence corporate performance as a result of managerial choices [70]. Using a system like EMA, organizations can collect, aggregate, and assess environmental data [71].

EMA systems serve as a decision-making support tool, facilitating organizations in enhancing both environmental and economic performance by detecting environmental costs and liabilities while supplying pertinent information to decision-makers and stakeholders [71]. Additionally, from a planning and control standpoint, EMA can provide information to implement and guarantee the success of environmental strategies by enabling effective monitoring, measurement, and evaluation of progress towards the attainment of corporate ecological and financial objectives [72]. To achieve these advantages, firms endeavor to establish environmental accounting and control systems while using environmental management practices [34].

An accounting system has to be appropriate for the circumstances in which it operates [38]. Vietnamese enterprises need to understand the extent to which use of the EMA information could help managers make non-routine decisions to improve environmental performance in the rapidly changing business environment of the transitional economy in Viet Nam. The benefits of EMA information have been identified by many studies [3,73]. However, in Vietnam as well as many other developing countries, the application of EMA is very limited [2,3].

### 2.6 Environmental performance

The term “environmental performance” describes a company’s degree of environmental effect management, environmental damage reduction, environmental responsibility performance, or the results of strategic environmental impact management actions [74]. Environmental performance is the result of how an organization manages its environmental variables, according to ISO 14031. Although there are many definitions of EP, most of them place an emphasis on the outcomes of environmental and activity-related management interventions. This is in conformity with ISO 14031’s definition of environmental performance. I argue that the ISO 14031 definition satisfies the requirements for a good definition as stated by Mansour, Alsulamy [75] since it is thorough and concise. It can be considered a broadly accepted term that contains significant aspects from prior academic definitions. ISO 14031 distinguishes two types of EP indicators: management performance indicators and operational performance indicators [76]. This study examines the management outcomes of top managers during the planning, strategy, and EMA phases. I’ll choose an EP scale that includes indicators of management performance.

The findings of EP research in one country may not be generalized to other countries- even though they are in the same region - due to differences in culture, economy, and the political and legal environments [77,78]. These differences may affect related factors that influence top managers’ implementation of GRS, choice of PES and use of the EMA information in organizations. Operating in a rapidly changing business environment, Vietnamese manufacturers need to understand the effects of related factors, such as green transformational leadership style, green reward systems, proactive environmental strategy, and top managers’ use of the EMA information on improve EP in the context of Vietnam.

Few studies examine (a) the impact of GTL style on choosing PES strategy, implementation of GRS and implementation of the EMA and EP; (b) the impact of choosing PES strategy on GRS implementation, implementation of the EMA and EP; and (c) the impact of GRS on EMA and EP. Moreover, there is little research into the relationship between EMA implementation on EP in a transitional economy such as Vietnam. Therefore, conducting a study on these relationships in Vietnam may help Vietnamese manufacturers gain a better understanding of the role of GTL style, PES strategy, GRS implementation, and implementation of the EMA in improving EP. This study focuses on examining the direct relationships through building and testing hypotheses H_1_ to H_10_ to answer RQ1, RQ2, RQ3 and RQ4.

### 2.7 Hypothesis development

#### 2.7.1 The impact of green transformational leadership on green reward system.

GTL reflects the attitudes and values of top management, significantly impacting the firm’s green human resource management [79]. GTL within a company plays a crucial role in the development of supportive green human resource management policies, facilitating the firm’s execution of its strategies and objectives to attain green performance [80]. Conversely, GRS encourages and enhances employee commitment to environmental responsibility and participation in eco-initiatives [8]. GTL would significantly contribute to the promotion of effective green human resource management techniques, such as GRS, by enabling top managers to inspire, stimulate, and encourage their followers to attain organizational environmental objectives [81].

H_1_: GTL style has a positive impact on GRS.

#### 2.7.2 The impact of green transformational leadership on proactive environmental strategy.

Based on UET, CEO ’s characteristics can influence strategic decisions made by firms [27], and transformational leadership is a significant trait of CEOs [82], indicating the antecedent role of CEO ‘s GTL in corporate PES adoption. CEOs’ green transformative leadership was found to be favorably associated to the firm’s PES [12]. CEO ‘s GTL not only uses idealistic influence and inspirational motivation to mold and alter a company’s values in order to satisfy environmental protection standards, but it also actively stimulates independent thinking and innovation to boost environmental performance. Previous research has shown that GTL is an important source of PES implementation [12,83,84].

H_2_: GTL style has a positive impact on PES.

#### 2.7.3 The impact of green transformational leadership on environmental management accounting.

Multiple studies have found that certain leadership styles influence the use of accounting information inside firms [53,85]. GTL leaders inspire and motivate employees to exceed environmental performance objectives and achieve targets [81]. Conventional accounting standards do not provide data appropriate for analyzing and monitoring environmental effect [86]. EMA is a practice that uses financial and non-financial data to improve a company’s environmental and economic performance and achieve long-term goals. It includes all accounting aspects influenced by an organization’s approach to environmental challenges and new eco-efficient practices [70], and it can help managers make decisions [33].

H_3_: GTL has a positive impact on EMA implementation of organizations.

#### 2.7.4 The impact of green transformational leadership on environmental performance.

GTL influences how employees perceive the company’s environmental performance in contrast to competitors in the same industry [87]. Multiple research have found a strong beneficial relationship between transformation leadership and organizational outcomes [88,89]. Green transformational leaders can improve perception, learning, integration, and coordination by challenging operational assumptions, articulating a clear vision, empowering employees, effectively inspiring, and encouraging team collaboration [55]. GTL improves green work engagement and environmental performance [81].

H_4_: GTL style has a positive impact on EP of organizations.

#### 2.7.5 The impact of proactive environmental strategy on green reward system.

A successful strategy requires an aligned control system [38]. Reward systems in Vietnam have evolved and developed; yet, there are significant variances amongst firms due to a variety of circumstances. In a quickly changing corporate environment, the impacts of contextual elements like strategy on reward systems are frequently [90]. GRS is a key component of green human resource management [29]. As one of the most important aspects of environmental management decisions, green human resource management is an important internal strategy to support the success of a PES [91].

H_5_: PES has a positive impact on GRS.

#### 2.7.6 The impact of proactive environmental strategy on environmental performance.

PES can provide competitive advantages through low-cost leadership and differentiation [92,93]. A distinctive company image, innovation, improved customer value, and increased efficiency make up the competitive differentiation advantage [47]. In contrast to its rivals, the PES promotes a distinctive corporate image [94]. Proactive environmental strategies and an organization’s success are positively correlated [95]. A company’s financial results can be enhanced by its environmental strategy and a particular PES that focuses on creating eco-friendly technologies [93]. According to a recent study, environmental policies have a good overall impact on environmental performance [4].

H_6_: PES has a positive impact on EP.

#### 2.7.7 The impact of proactive environmental strategy on environmental management accounting.

A proficient control and management system that corresponds with the organization’s strategy will substantially enhance the achievement of that strategy [38]. Wijethilake [96] contended that there exists a favorable correlation between proactive environmental strategies and sustainability control systems. Environmental strategies, as components of an eco-control package, can enhance both the environmental and economic performance of enterprises through the application of EMA [97]. Latan, Jabbour [34] discovered in research of Indonesian listed corporations that organizational green resources, such as environmental strategies, enhance companies’ environmental performance through the utilization of EMA.

H_7_: PES has a positive impact on EMA.

#### 2.7.8 The impact of the green reward system on environmental performance.

GRS is a crucial element of green human resource management [98], and green human resource management positively influences the organization’s environmental performance [99]. The GRS will be employed to foster green innovation and progress by encouraging personnel to contribute inventive ecological ideas pertinent to their respective roles [100]. This approach can persuade employees to adopt eco-friendly behaviors instead of fostering negative behaviors [101]. The implementation of sustainable initiatives may be integrated into employee reward systems to incentivize behavioral change and enhance overall performance [102].

H_8_: GRS has a positive impact on EP.

#### 2.7.9 The impact of the green reward system on environmental management accounting.

Green human resources management encompasses eco-friendly recruitment, training, and reward systems to promote environmental protection and effectively implement sustainability objectives [66]. Green rewards alone may not result in substantial environmental improvement; nevertheless, when combined with feedback, empowerment, and clear communication, they can facilitate environmental advancements [103]. The EMA is ensuring that enterprises effectively utilize available green human resources to promote environmentalism [104]. Currently, EMA advocates for enhanced operational efficiency to optimize corporate environmental governance and reduce operational expenses, alongside comprehensive cost accounting, benefits evaluation, and strategic environmental management planning.

H_9_: GRS has a positive impact on EMA.

#### 2.7.10 The impact of environmental management accounting on environmental performance.

EMA can help firms satisfy their environmental duties while also discovering the financial benefits of increased environmental and economic effectiveness [105]. Implementing EMA allows managers to optimize resource use while also improving environmental performance [33]. The use of EMA has the potential to improve an organization’s environmental performance by allowing it to understand the complexities of its environmental management systems and make more informed decisions [106]. Both businesses were formed with the goal of attaining corporate objectives and long-term environmental benefits [107]. Prior research showed that implementing EMA can increase a company’s environmental performance [67].

H_10_: EMA implementation has a positive impact on EP.

Fig 2 illustrates the proposed research model by the author.

## 3 Research methodology

### 3.1 Research design

The research design is demonstrated with several aspects, including research type, research strategy, research philosophy, and research strategy, in order to address the research questions [108]. The objective of this study is to describe specific events or situations and to elucidate the relationships between variables, which is why it could be classified as a descriptive-explanatory study in several research types [108]. The study specifically investigates the causal relationships between factors: the GTL, GRS, PES, and using EMA information by top managers and the EP of Vietnamese manufacturers. In order to gain a more comprehensive understanding of these relationships and to provide an explanation, it is necessary to compile both quantitative and qualitative data for statistical tests. This study is influenced by a philosophy that is closely related to positivism, which is characterized by the use of extant theories to develop hypotheses. The hypotheses are subsequently tested and either confirmed or rejected, which results in the further development of the theories in future research [108]. The present investigation implements a deductive methodology. Several primary characteristics of this approach are mentioned by Saunders, Lewis [108]: (1) to elucidate causal relationships between variables by formulating and testing hypotheses; (2) to guarantee reliability by employing highly structured methodologies; (3) to operationalize concepts in a manner that enables quantitative measurement of facts. The deduction approach is employed in conjunction with the survey strategy in this investigation [108]. There are numerous reasons why this approach is highly favored in the field of business and management research: (1) to enable the collection of data from a large sample size; (2) to standardize the data through a questionnaire, resulting in a comparative ease of explanation and comprehension; (3) to enable the collection of quantitative data for quantitative descriptive analysis. The mixed method is implemented in this study. This method can leverage the benefits of both quantitative and qualitative methodologies: (1) to generalize the results to a specific population and (2) to offer a comprehensive understanding of the context [109].

### 3.2 Variables measurement

This study adopted the scales from prior research. Singh, Del Giudice [5] conducted a study on GTL, which included six components. The GRS scale comprises three items, as established by Masri [110]. The PES scale was derived from a study by Dai, Cantor [36] and has five elements. The EMA scale is derived from a study conducted by Chaudhry and Amir [106] and comprises six items. The EP scale comprises seven items and is founded on the research conducted by Lisi [111] and Yang Spencer, Adams [112]. All items utilized a 5-point Likert scale, where 1 represents strongly disagree and 5 represents strongly agree. The measurement scales are detailed in Table 2.

**Table 2:** Construct reliability and validity.

Research constructs/Items	Outer Loadings	Α	RhoA	C.R	AVE
**Green Transformational Leadership** (Singh et al., 2020)					
•** GTL1:** I motivate subordinates through the implementation of an environmental plan.	0.952	0.934	0.942	0.949	0.846
•** GTL2:** I impart a distinct environmental vision to my subordinates.	0.932				
•** GTL3:** I actively urge my subordinates to contribute to the development of an environmental plan.	0.921				
•** GTL4:** I promote the achievement of environmental objectives among employees.	0.930				
•** GTL5:** I take into account the environmental opinions of my subordinates.	0.956				
•** GTL6:** I encourage subordinates to explore and contribute their environmentally friendly ideas.	0.955				
**Green reward system (**Masri, 2016)					
•** GRS1:** Environmental performance is acknowledged publicly through awards, banquets, and media coverage.	0.812	0.709	0.706	0.823	0.609
•** GRS2:** The company gives non-monetary benefits for environmental work, such as holidays, paid time off, gifts, bonuses, cash, premiums, and promotions.	0.802				
•** GRS3:** Connect suggestion systems to reward systems by giving awards for new environmental actions or work.	0.724				
**Proactive environmental strategy** (Dai, Cantor, & Montabon, 2017)					
•** PES1:** Our company consistently endeavors to exceed the minimum requirements of environmental laws and regulations.	0.755	0.812	0.832	0.868	0.571
•** PES2:** The top managers in our organization prioritize environmental concerns significantly.	0.619				
•** PES3:** Our firm is at the forefront of environmental challenges within our sector.	0.753				
•** PES4:** The environmental risks that have an impact on our business are successfully managed by our organization.	0.784				
•** PES5:** Environmental issues are a top focus for our business management.	0.848				
**Environmental management accounting** (Chaudhry et al., 2020)					
•** EMA1:** The system of accounting employed by our organization to document all tangible inputs and outputs, encompassing energy, water, materials, trash, and emissions.	0.822	0.833	0.849	0.877	0.546
•** EMA2:** The organization’s accounting system can analyze product inventories, product improvements, and product environmental effects.	0.806				
•** EMA3:** Environmental performance goals are used by our company for both tangible inputs and results.	0.754				
•** EMA4**: Our company’s accounting system is capable of identifying, estimating, and classifying environmental expenses and liabilities.	0.740				
•** EMA5:** Our company’s accounting system can create and use cost accounts relating to the environment.	0.685				
•** EMA6**: Our company’s accounting system offers the capacity to allocate environmental costs to specific items as well as environmental costs to products.	0.602				
**Environmental performance** (Lisi, 2015; Yang Spencer et al., 2013)					
•** EP1:** Adhering to environmental regulations	0.760	0.847	0.852	0.884	0.522
•** EP2:** Preventing and alleviating environmental disasters.	0.685				
•** EP3:** Identifying potential avenues for reducing costs.	0.714				
•** EP4:** Environmental impact reduction beyond	0.621				
•** EP5:** Enhanced reputation.	0.753				
•** EP6:** Creating societal advantages	0.725				
•** EP7:** Enhanced competitive edge	0.788				

### 3.3 Participants and sample size

This study selected the CEOs of manufacturing companies in Vietnam as this study ‘s subjects. CEOs are seen as the preeminent leaders inside firms, playing a vital role in dealing with employee concerns [113]. They have comprehensive knowledge of every aspect of the organization [114]. The manufacturing sector is regarded as the foremost catalyst of the economy [115]. In the organizational environment of Vietnam, the manufacturing sector significantly contributes to economic stability through big and medium enterprises [116]. The manufacturing sector accounts for 33.10% of employment generation in Vietnam [117]. It also facilitates swift technical advancement and provides convenient access to worldwide manufacturing networks [118]. In economic growth, it is the predominant sector, contributing 35.47% to the nation’s total GDP in 2023 [117]. Nonetheless, similar to other countries (e.g., China, India, Russia, and South Africa), Vietnam’s industrial sector experiences a boom-bust cycle, rendering it unsustainable. Consequently, I chose the manufacturing sector as the foundation for this study. A total of 2,500 surveys I distributed via direct contact and emails, resulting in the return of 255 raw samples, providing a response rate of 10.2%. The data has been cleaned and properly evaluated. Following all checks (i.e., for missing values and outliers), 198 acceptable samples were retained for the final analysis.

### 3.4 Survey tools and distribution modes

A cross-sectional survey was done due to its lower cost and complexity compared to a longitudinal survey. Van der Stede, Young [119] assert that the results of a cross-sectional survey method may diminish confidence. Consequently, a robust theoretical foundation was established by a comprehensive literature assessment. Variables measurement from prior studies were used to assess the research variables, enhancing reliability and validity. Furthermore, essential factors (e.g., logical sequence, recognizable terminology, unambiguous response format) were considered in the creation of the questionnaire to minimize response errors. The final version was developed and utilized for data collection.

A survey questionnaire was employed to collect data to assess the association between the variables [120]. The utilization of the questionnaire is a suitable method and one of the most effective instruments in social science research. The necessary elements were derived from the academic literature, aiding researchers in examining and assessing the validity and dependability of previously investigated theories and hypotheses [121]. I executed a pilot study to assess the validity and reliability of the research instrument (the questionnaire) to ensure its accuracy [122]. The overall reliability was determined to exceed 0.60, deemed an exceptional outcome. The questionnaire’s validity was affirmed by university professors who are specialists in the field. I utilized a convenience sample method for choosing participants. The questionnaire is utilized to acquire extensive samples. This technique is effective and facilitates straightforward sample assembly. I supplied participants with survey instructions and assurances of confidentiality and privacy, obtaining their agreement while meticulously adhering to ethical norms to safeguard their rights.

The results of the statistical sample in Table 1 show that the textiles, leather, and shoes industries account for a high proportion in the manufacturing industry (40.0%). The age of the respondents is concentrated mainly over 30 years old (93.0%) in the sample, showing that the respondents have enough experience in the process of managing work to answer the survey. The educational background with a postgraduate degree accounts for a significantly higher proportion (62.5%). The time the company was established for over 6 years accounts for a high proportion. With the characteristics of CEOs and businesses, it is possible to ensure a relatively representative representation of the population.

**Table 1:** Demographics of the participating firms and respondents.

Tenure	Freq.	%	Gender	Freq.	%
1-5 years	29	15.0	Male	135	68.0
6-10 years	63	32.0	Female	63	32.0
11-15 years	40	20.0	**Total**	**198.0**	**100.0**
15-20 years	44	22.0	**CEO’s Education**	Freq.	%
>20 years	22	11.0	Pre-undergraduates	5	2.5
**Total**	198.0	**100.0**	Graduates	69	35.0
**Type of manufacturing**	**Freq.**	**%**	Post-graduates	124	62.5
Textiles, leather, and shoes	79	40.0	**Total**	198.0	**100.0**
Plastic, packaging	30	15.0	**Founded time of firms**	**Freq.**	**%**
Mechanical machines	10	5.0	< 5 years	14	7.0
Pharmaceutical	18	9.0	6–10 years	88	45.0
Process the wood	24	12.0	11-25 years	64	32.0
Food production	4	2.0	> 25 years	32	16.0
Other production	33	17.0	**Total**	198.0	**100.0**
**Total**	198.0	**100.0**	**Questionnaires**	**Freq.**	**%**
**CEO’s age**	**Freq.**	**%**	Sent	2,500	
18-29	14	7.0	Received	255.0	
30-49	79	40.0	Missing value	6	
50-64	75	38.0	Excluding small firm	51	
>64	30	15.0	Final sample	**198.0**	
**Total**	198.0	**100.0**			

Source: Compiled by the author from collected data

To obtain the official results through the use of SmartPLS3 software, two PLS-SEM model evaluation processes were performed: (1) measurement model assessment and (2) structural model assessment [123]. Measurement model assessment includes many different evaluations: (1) Internal consistency evaluation based on composite reliability, (2) Convergent validity evaluation, considering the individual reliability of each scale (outer loading) and average variance extracted (AVE) and (3) using additional Fornell-Larker criteria and Cross loading, HTMT (discriminant validity) criteria were also considered. Evaluation of structural models includes the following steps: The assessment of multi-collinearity (VIF), Evaluation of significance of hypotheses, R^2^, f^2^, Q^2^.

## 4. Results

Data analysis in this investigation is conducted by using SEM-PLS, specifically Version 3 of SmartPLS [124]. Partial least squares (PLS) are validated by their suitability for causal-predictive analysis of complex models that include multiple independent and dependent variables [125]. In addition, it is important to mention that PLS is well-suited for small sample sizes, imposes minimal restrictions at measurement levels, and does not require multivariate normal data [126].

### 4.1. Measurement model

The average variance extracted (AVE), composite reliability (CR), and Cronbach alpha (α) values for each latent variable are displayed in Table 2. Furthermore, the convergent validity is substantiated by the α, CR, and AVE values also displayed, as they exceed the preset threshold values (α > 0.7, CR > 0.7, and AVE > 0.5), which were established by Fornell and Larcker [127] and Hair, Hollingsworth [123]. This measure indicates the dependability of the items that are loaded onto each construct. All factor loadings were above the threshold of 0.7. The data consistency was assessed using Dijkstra-Henseler’s rho (rho_A) [128], which provides additional evidence of the reliability of the items put on each construct. The results obtained from the structural model were deemed satisfactory.

The evaluation of discriminant validity was performed using the Fornell and Larcker criterion, cross-loadings, and the Heterotrait–Monotrait ratio of correlations (HTMT) criterion as specified by Hair, Hollingsworth [123]. Table 3 presents the square roots of the average variance extracted (AVE) values derived from the correlation matrix. The diagonal values of the correlation matrix for the latent variables were observed to be greater than or equal to the diagonal values below them, providing convincing proof for the assumption of discriminant validity.

**Table 3:** Fornell-Larcker Criterion.

Constructs	Environmental management accounting	Environmental performance	Green reward system	Green transformational leadership	Proactive environmental strategy
Environmental management accounting	0.739				
Environmental performance	0.467	0.723			
Green reward system	0.423	0.517	0.780		
Green transformational leadership	0.300	0.551	0.387	0.941	
Proactive environmental strategy	0.413	0.363	0.357	0.146	0.756

Table 4 displays HTMT values that fall below the established threshold of 0.90, as indicated by Hair et al. (2017), providing evidence of the presence of discriminant validity.

**Table 4:** Heterotrait-Monotrait Ratio (HTMT).

Constructs	Environmental management accounting	Environmental performance	Green reward system	Green transformational leadership	Proactive environmental strategy
Environmental management accounting					
Environmental performance	0.530				
Green reward system	0.542	0.673			
Green transformational leadership	0.301	0.603	0.465		
Proactive environmental strategy	0.476	0.418	0.470	0.153	

### 4.2. Structural model

Before evaluating the structural correlations, it is essential to test for collinearity to avoid potential bias in the regression outcomes. Variance inflation factor (VIF) values over 5 indicate probable collinearity issues among the predictor variables. It is important to acknowledge that collinearity issues may also occur at lower VIF values between 3 and 5 [124], 2015). All values in this study are under 5. The coefficient of determination (R^2^) quantifies the amount of variance accounted for in each of the endogenous constructs, serving as an indicator of the model’s explanatory capacity. According to Hair et al. (2017), R^2^ values of 0.75, 0.50, and 0.25 can be classified as strong, moderate, and weak, respectively. The R^2^ values for the GRS, PES, EMA implementation, and EP in the model are 0.242, 0.21, 0.279 and 0.475, respectively.

To evaluate R^2^, the significant impact size (f^2^) is utilized. The process of excluding a certain independent variable from the study framework and then analyzing its subsequent effects on the dependent variable is known as exclusion. Hair, Hollingsworth [123] recommend comparing f^2^ values with thresholds of 0.02 (weak), 0.15 (moderate), and 0.35 (strong) to determine the strength of the effects of independent variables. Based on the results, it can be observed that all variables with f^2^ coefficients are greater than 0.15, demonstrating a significant influence on environmental performance.

To increase the predictive accuracy of the R^2^ value, researchers also added another criterion, which is the Q^2^ value. This measurement is an indicator of out-of-sample predictive power in the model. The values 0.02, 0.15 and 0.35 of R^2^ represent low, medium and high predictability, respectively. The respective Q^2^ values for GRS, PES, EMA, and environmental performance are 0.144, 0.172, 0.185 and 0.208. According to Hu and Bentler [129], the SRMR is considered an absolute measure of fit, where a value of zero indicates a perfect fit. The current value of this figure in this study is approximately 0.06, which aligns with the range suggested by Hu and Bentler [129]: the SRMR value in the PLS-SEM approach should be below 0.08 or 0.10.

**Fig 3:**
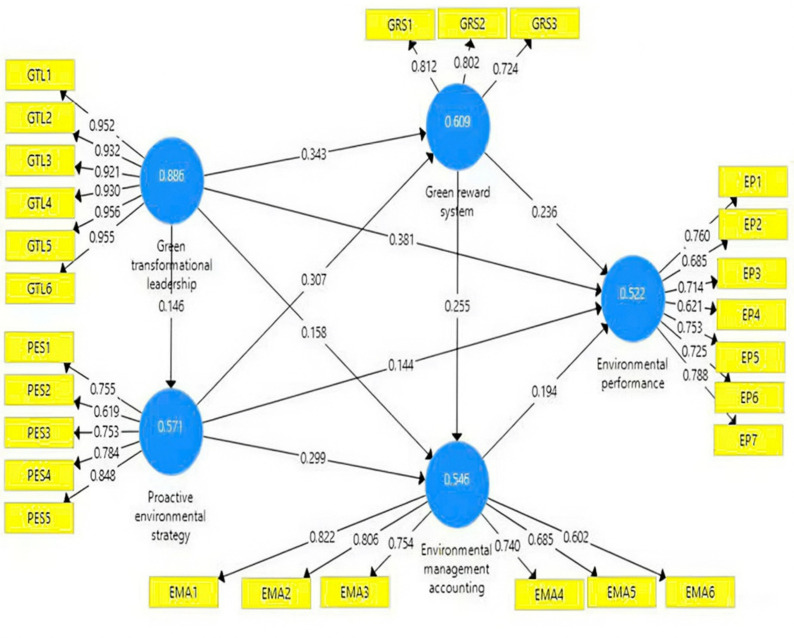
PLS-SEM analysis results of the theoretical model.

This study conducted PLS-SEM analysis to investigate the hypotheses proposed in this study (Fig 3). Table 5 displays the outcomes of the estimation conducted for the structural equation model.

**Table 5:** Hypothesis testing.

Relationships	Original Sample (O)	Sample Mean (M)	Standard Deviation (STDEV)	p-values	Results
H_1_: GTL - > GRS	0.343	0.341	0.074	0.000	Accepted
H_2_:GTL - > PES	0.146	0.146	0.071	0.040	Accepted
H_3_:GTL - > EMA	0.158	0.151	0.077	0.041	Accepted
H_4_:GTL - > EP	0.381	0.375	0.076	0.000	Accepted
H_5_:PES - > GRS	0.307	0.321	0.065	0.000	Accepted
H_6_:PES - > EP	0.144	0.145	0.065	0.028	Accepted
H_7_:PES - > EMA	0.299	0.296	0.077	0.000	Accepted
H_8_:GRS - > EP	0.236	0.241	0.110	0.033	Accepted
H_9_:GRS - > EMA	0.255	0.268	0.090	0.005	Accepted
H_10_: EMA - > EP	0.194	0.194	0.083	0.020	Accepted

## 5 Discussion

The results shown in Table 5 demonstrate that GTL exerts a statistically significant and advantageous effect on GRS, PES, EMA implementation, and EP (p-value < 0.05). This outcome further corroborated the initial proposal of Hambrick and Mason [27]. GTL influences both the design and operation of an administrative system (GRS) as well as the implementation of a strategy (PES). The top management shapes the creation of a comprehensive administrative system for the firm, and a GRS plays a crucial role in inspiring employees and assisting them in recognizing their significant contribution to environmental management. The GTL of CEOs uses idealized influence and inspirational motivation to shift corporate values in alignment with environmental preservation, accomplished through the adoption of PES. This result corroborates the studies of Jia, Liu [80] and Sharin, Hanafi [84]. The EMA is seen as a system that delivers information pertinent to the environment, aiding environmental performance indicators. The results corroborate prior studies indicating a significant association between high-level management ‘s GTL and the application of EMA in organizations [85]. This outcome aligns with the findings of Singh, Del Giudice [5] and Hanif, Ahmed [3], who identified that the green transformational leadership (GTL) of top managers is a factor that can enhance green innovation and improve the environmental performance of firms. Consequently, hypotheses H_1_, H_2_, H_3_ and H_4_ are supported.

Moreover, the study demonstrated a strong and favorable association between the PES and the adoption of both GRS, EP, and EMA (p-value < 0.05), hence supporting hypotheses H_5_, H_6_ and H_7_. A good strategy requires support from control systems [38]. A company with a PES may utilize its tangible and intangible resources effectively, resulting in a minimized environmental impact. These concerns are resolved with GRS and EMA. The green reward system encompasses all economic and non-economic incentives offered to employees to attract, motivate, and involve them in achieving organizational environmental objectives. This result corroborated the findings of Daily, Bishop [91]. Furthermore, the application of EMA is seen as a solution that aids managers in making informed decisions in unpredictable environments. EMA provides information that results in indicators for environmental performance. This result reinforces the findings of Latan, Jabbour [34], who demonstrated that environmental strategies enhance corporate environmental performance through the utilization of EMA, and aligns with the research of Kraus, Rehman [4], which indicated that an environmental strategy facilitates improvements in environmental performance within companies.

The deployment of GRS significantly and positively impacts on EP and EMA, as evidenced by a p-value < 0.05. This finding corroborates the research of Jabbour and de Sousa Jabbour [102], which indicated that a firm can enhance its overall performance by providing green rewards to its staff. GRS can incentivize employees to adopt environmentally sustainable practices and encourage green behaviors. Moreover, green rewards alone may not substantially improve environmental performance; nevertheless, when combined with feedback, empowerment, and clear communication, they can facilitate environmental advancements. Currently, EMA encourages efficiency in operations to enhance organizational environmental performance and reduce operating expenses, thereby increasing profitability through comprehensive cost accounting, benefits evaluation, and environmental management establishing. This result is supported by Peng, Tu [83], Govindarajulu and Daily [103] Consequently, the results supported hypotheses H_8_ and H_9_.

The findings indicate that the implementation of EMA positively influences EP, therefore confirming hypothesis H_10_. EMA assists organizations in effectively attaining their environmental, economic, and financial objectives. Moreover, when firms reveal their environmental policies in financial statements, it cultivates a favorable perception among stakeholders and draws additional investors. This result corroborated the studies of Asiaei, Bontis [67].

## 6 Conclusion and implications

### 6.1 Conclusion

The top manager’s GTL style can both motivate and aid employees in the development of these competencies and in their active participation in the organizational objective to improve corporate environmental performance. Organizations can currently accomplish environmental sustainability by enhancing their internal capabilities. The design, implementation, and operation of complex administrative systems such as GRS and EMA, as well as the selection and pursuance of an environmental strategy such as PES, are significantly influenced by CEOs ‘s leadership style of manufacturing enterprises in Vietnam. This research demonstrates that the GTL of CEOs must be integrated with a variety of strategies and control systems within the organization, and these resources must be in proper alignment with one another. Afterward, it contributes to the improvement of environmental performance. This outcome underscores the significance of EMA implementation in order to improve organizational environmental performance, as organizations are primarily focused on enhancing their financial performance. EMA, on the other hand, enhances organizational environmental performance without compromising financial performance [130]. EMA is a term that refers to the administration of financial and non-financial information by organizations in order to evaluate the repercussions of their decisions to enhance environmental performance [34].

### 6.2 Theoretical implications

This research contributes to the existing body of literature in numerous ways. Initially, the theoretical framework for the development of my research model is improved by the inclusion of UET. This study addresses the necessity of conducting research that integrates GTL, GRS, PES, EMA, and EP, as recommended by Liem and Hien [2] and Hiebl [44]. In addition, this investigation addresses the necessity of overcoming the behavioral perspective to gain a more comprehensive understanding of the effects of GTL on strategy, control systems, and performance, as recommended by Hambrick and Mason [27]. Secondly, the prevailing behavioral perspective in the current collection of literature on CEOs’ GTL is advantageous in that it improves the capabilities, incentives, and opportunities of employees. However, it fails to provide a comprehensive understanding of the internal mechanisms of a business in its pursuit of environmental performance objectives. The UET framework provides an explanatory framework that functions as an alternative to the behavioral perspective, with the objective of elucidating the role of top management in the connection between various resources. The results of this investigation underscore the importance of four critical organizational resources: GTL, GRS, PES, and EMA implementation, in enabling the manufacturers to optimize their environmental performance. Lastly, this research enhances the current body of knowledge on GTL by investigating the role of top leaders in the collection of information regarding the effects of GTL. Until recently, leadership has evolved independently, with the goal of influencing individuals to attain an organization’s strategic objectives. My research demonstrates an additional form of leadership that is relatively underexplored and can be derived from CEOs. Scholars have argued that the endorsement of CEOs and other high-ranking executives is essential for extracting value from PES and EMA [9]. This implies that the effects are optimized when the fundamental values of GRS, PES, and EMA are consistent with those of top leaders [2,27].

### 6.3 Managerial implications

In the practical implications, these findings provide a deep understanding of how companies certified by ISO 14001 in Vietnam should improve their environmental performance by developing leadership style to the environment, designing GRS properly, implementing suitable environmental strategies, and using EMA information. Many organizations in developing nations, such as Vietnam, are still unable to actively contribute to environmental stability and are not using EMA to improve their environmental effect. This finding might serve as a guide for organizational decision-makers looking to enhance environmental performance over time. Other practical implications of the research for top managers in Vietnamese manufacturing enterprises recognize the importance and usefulness of GRS and EMA, which help them be motivated to establish and build these control systems during the implementation of the organization’s environmental strategy.

### 6.4 Limitations and directions for future research

This study also has a number of limitations that future scholars can study. First, top managers’ leadership styles take many different forms, while this study focuses only on GTL. Therefore, future studies need to explore this aspect. Secondly, this is a cross-sectional study, so it requires long-term research over time to be able to compare its environmental performance before, during, and after the implementation of GRS, PES and EMA. Finally, future research needs to measure the others performance (financial, competitive advantage …)

## Supporting information

Data 1 S1 Data. (XLS)
